# The molecular entities in linked data dataset

**DOI:** 10.1016/j.dib.2020.105757

**Published:** 2020-05-27

**Authors:** Dominik Tomaszuk, Łukasz Szeremeta

**Affiliations:** Institute of Informatics, University of Białystok, 15-245 Białystok, Poland

**Keywords:** Linked Data, Semantic Web, RDF, JSON, Cheminformatics, molecular dataset

## Abstract

The Molecular Entities in Linked Data (MEiLD) dataset comprises data of distinct atoms, molecules, ions, ion pairs, radicals, radical ions, and others that can be identifiable as separately distinguishable chemical entities. The dataset is provided in a JSON-LD format and was generated by the SDFEater, a tool that allows parsing atoms, bonds, and other molecule data. MEiLD contains 349,960 of ‘small’ chemical entities. Our dataset is based on the SDF files and is enriched with additional ontologies and line notation data. As a basis, the Molecular Entities in Linked Data dataset uses the Resource Description Framework (RDF) data model. Saving the data in such a model allows preserving the semantic relations, like hierarchical and associative, between them. To describe chemical molecules, vocabularies such as Chemical Vocabulary for Molecular Entities (CVME) and Simple Knowledge Organization System (SKOS) are used. The dataset can be beneficial, among others, for people concerned with research and development tools for cheminformatics and bioinformatics. In this paper, we describe various methods of access to our dataset. In addition to the MEiLD dataset, we publish the Shapes Constraint Language (SHACL) schema of our dataset and the CVME ontology. The data is available in Mendeley Data.

Specifications Table

**Table:** 

Subject	Computer science (Information Systems)
Specific subject area	Semantic Web, Linked Data
Type of data	Graph
How data were acquired	Document data was acquired by fetching available public domain documents and generated by a software.
Data format	Raw Filtered JSON-LD (RDF)
Parameters for data collection	Total of ‘small’ chemical entities (349,960), total of data RDF triples (7083,730), total of SHACL RDF triples (89), total of ontology RDF triples (59).
Description of data collection	Data, stored in different schemas and formats (e.g. SDF, SMILES, InChI), was processed and merged where duplicates were found using matching criteria on chemical formula (including the structural formula and the molecular formula) and other identifiers (URL, InChI) and the document was extended accordingly.
Data source location	Institute of Informatics, University of Bialystok Konstantego Ciolkowskiego 1 M, 15–245 Bialystok
Data accessibility	Repository name: Mendeley Data Data identification number: https://doi.org/10.17632/fp4phyrbkz.1 Direct URL to data: https://data.mendeley.com/datasets/fp4phyrbkz/1

## Value of the data

• The presented dataset of molecular entities is useful because it includes a classification, whereby the relationships between molecular entities and their parents and/or children are described.
• The provided dataset is also useful, because all chemicals in the dataset contain a subsumption relationship, meaning that all of the molecular entries are available to semantic reasoning tools that harness the classification hierarchy.
• The dataset may be beneficial for people concerned with research and development tools for cheminformatics and bioinformatics.
• The dataset may be also beneficial for the users of information services and systems, along with those who use them through query or inference operations.
• The dataset can be used for further research on searches to be expanded and redefined, or present references to resources with content related to that of those directly retrieved in the search, or suggest new search terms.
• An additional value of the dataset is that it is sharable, extensible, and easily re-usable. Resources can be described in collaboration with other datasets and linked to data contributed by other communities.

## 1 Data description

**Fig. 1:**
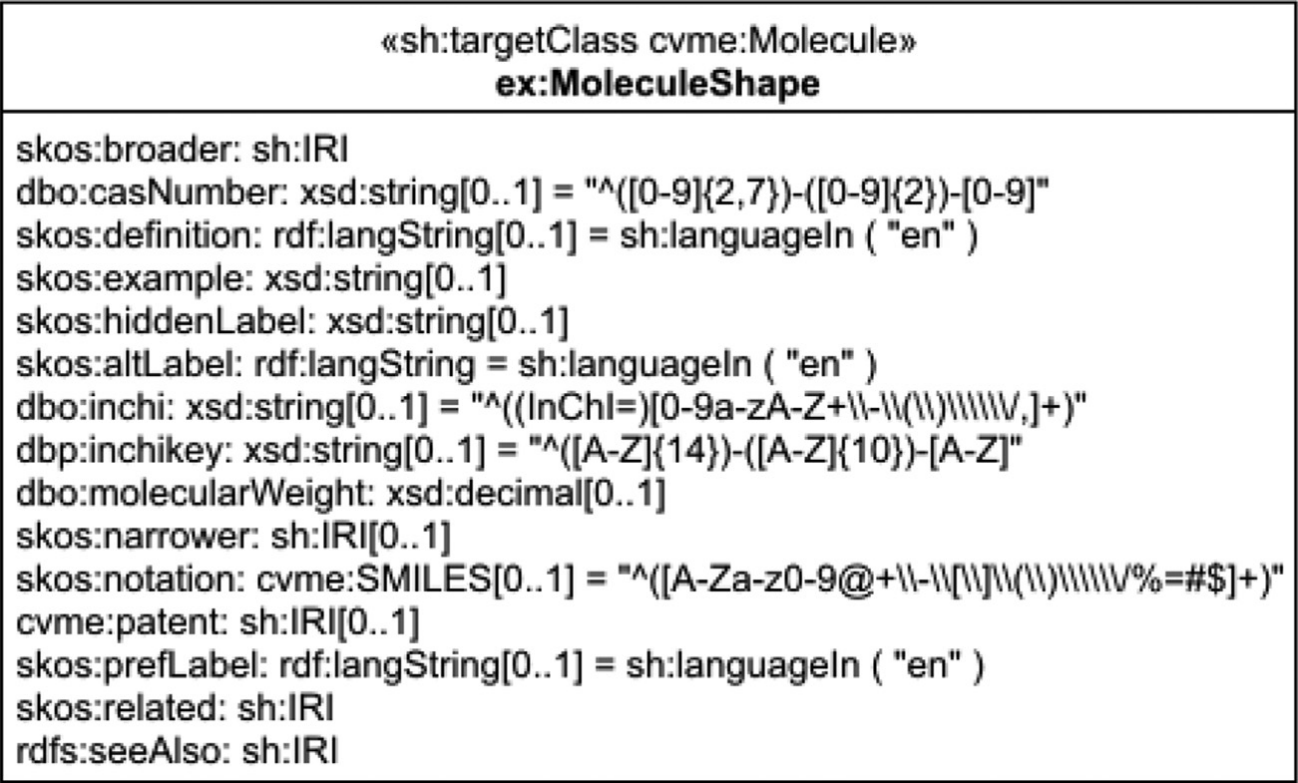
SHACL schema in UML notation.

The Molecular Entities in Linked Data (MEiLD) dataset comprises data of distinct atoms, molecules, ions, ion pairs, radicals, radical ions, and others that can be identifiable as separately distinguishable chemical entities. As a basis, the dataset uses the Resource Description Framework (RDF) data model [1,2]. To describe chemical molecules, vocabularies such as Chemical Vocabulary for Molecular Entities (CVME) and Simple Knowledge Organization System (SKOS) [3] were used. The first one is the designed vocabulary for this dataset, and the second is a well-known way to represent structured controlled vocabularies. Other well-known ontologies and vocabulary, e.g. DBPedia Ontology [4], were also used for interoperability. The schema of our dataset is described in SHACL [5] and encoded in JSON-LD format [6]. Fig. 1 presents the schema in UML notation. The CVME vocabulary for our dataset is encoded in OWL [7].

The CVME vocabulary for our dataset is written in Web Ontology Language (OWL). In CVME, molecular entities are modeled as instances of the class cvme:MolecularEntity, which is a subclass of skos:Concept. This way, interoperability with other SKOS vocabularies and standard tools is maintained. The different names of the entity are represented using the label properties of SKOS: skos:prefLabel for the IUPAC name, skos:altLabel for alternative names, skos:hiddenLabel for molecular formula (without subscripts). A textual description of the molecular entity is expressed using skos:description. The different line notation encodings of the entity can be represented using skos:notation property, with the datatype of the notation string expressing the specific format used (e.g., SMILES, InChI, etc.). The connection table formats are encoded in skos:example properties.

**Fig. 2:**
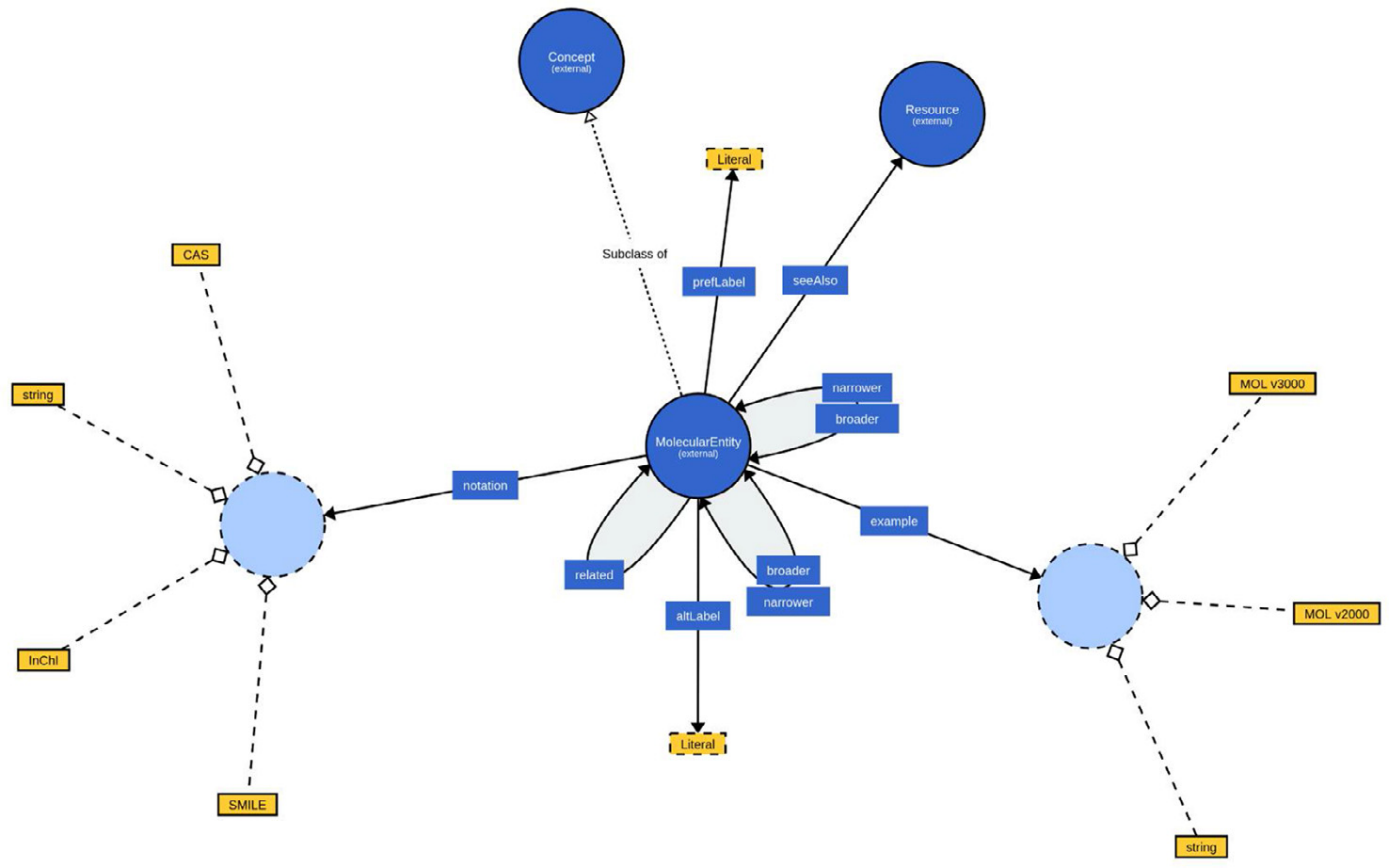
CVME vocabulary in VOWL notation.

For mapping the molecular entities to similar entities in external data sources, several properties are used for different purposes, property rdfs:seeAlso provides contextual information about the molecular entity for humans (e.g., web page). The properties skos:broader and skos:narrower are used to assert a hierarchical link between two molecular entities, ie. defines functional parents, shows parts of entities. The property skos:related is used to assert a role link between two chemicals. The vocabulary in VOWL notation [8] is shown in Fig. 2.

In the remainder of the paper, we present and discuss multiple options for access to MEiLD. We use prefixes to replace full namespaces in IRIs to keep them more readable. Instead, we provide the full list of the prefixes used and their corresponding namespaces in Table 1.

**Table 1:** Prefixes used in the dataset.

Prefix	Namespace
rdf	http://www.w3.org/1999/02/22-rdf-syntax-ns#
rdfs	http://www.w3.org/2000/01/rdf-schema#
owl	http://www.w3.org/2002/07/owl#
skos	http://www.w3.org/2004/02/skos/core#
cvme	http://ii.uwb.edu.pl/cvme#
xsd	http://www.w3.org/2001/XMLSchema#
dbp	http://dbpedia.org/property/
dbo	http://dbpedia.org/ontology/
ex	http://example.com/

## 2 Experimental design, materials, and methods

### 2.1 Dataset preparation

The dataset is in JSON-LD format and was generated by the SDFEater tool.^1^ JSON-LD is a format used in the Semantic Web [9] world to serialize data in the RDF model. Saving the data in such a model allows preserving the semantic relations between them. These relations can be hierarchical or associative. The first one indicates that a molecular entity is more general or more narrower than other. The second relation defines that the two molecular entities are inherently related. SDFEater is a Java-based, multiplatform, Command-Line Interface (CLI) program that allows parsing atoms, bonds, and other molecule data placed in chemical data Structure-data file (SDF) format. The tool supports a number of different output RDF formats including Turtle, N-Triples, and RDFa. Our dataset is based on the CheBI^2^ SDF file available under the Creative Commons Attribution 4.0 International license^3^ and enriched with additional ontologies. The MEiLD dataset also contains line notation data eg. Simplified Molecular-Input Line-Entry System (SMILES) and International Chemical Identifier (InChI). JSON-LD is based on the JSON format. JSON stands out in terms of machine processing performance and human readability. JSON-LD allows keeping all information about RDF triples and their relationships.

### 2.2 Data sources

To enable data from different sources to be connected and queried we enrich a MEiLD dataset with links to other data to provide a context. For the best principles for publishing and interlinking structured data over the Web [10], our dataset meets the following rules:

1. use IRIs as names for things,
2. use HTTP IRIs so those names can be looked up (dereferencing),
3. return useful information upon lookup of those IRIs (especially RDF),
4. include links by using IRIs that dereference to remote documents.

Our dataset support also a five-star Linked Data scheme for Web publishing [11], in particular:

1. our dataset is published under an open license,
2. our dataset is published as structured data,
3. we use non-proprietary formats,
4. we use IRIs to identify things,
5. we link our data to other data.

All links that were embedded in a MEiLD dataset are listed in Table 2. Due to the fact that the dataset is in both RDF and JSON formats, different methods of data access are possible. We present them below.

**Table 2:** Cross-referenced sources in a MEiLD dataset.

Name	URL
ChEBI	https://www.ebi.ac.uk/chebi/
KEGG	http://www.genome.jp/
PubChem Compound	https://pubchem.ncbi.nlm.nih.gov/compound/
PubChem Substances	https://pubchem.ncbi.nlm.nih.gov/substance/
Rhea	https://www.rhea-db.org/
UniProt	https://www.uniprot.org/

### 2.3 Access method 1: path-based access

A MEiLD dataset uses JSON-LD syntax. Like any JSON document, our dataset can be processed using various tools to navigate around the tree, selecting nodes by a variety of criteria.

JSONPath^4^ is one of such approach. It is based on the syntax known from XPath. It supports JSON documents (including JSON-LD). For example, the JSONPath query

$['@*graph*'][?(@.molecularWeight<400)]['*inchi*']['@value'] returns the International Chemical Identifier (InChI) of molecules with molecular weight less than 400.

$['@*graph*'][?(@.molecularWeight<400)]['*inchi*']['@value']

Another tool is jq.^5^ It is based on the text processing command syntax known from UNIX systems such as sed, awk and grep. For example, the iq query

if .[]."http://www.w3.org/2004/02/skos/core#narrower" | length == 0 then "no narrower terms" else "more than zero narrower terms" returns information about the narrower terms.

### 2.4 Access method 2: RDF APIs

Because our dataset uses JSON-LD which is an RDF serialization, it is also possible to process it as an RDF document. There are many publicly available programming libraries and tools for this purpose. Among them, we can distinguish simpler libraries such as RDFLib written in Python and Redland RDF Library (librdf) written in C, as well as powerful tools with support for inference mechanisms such as Apache Jena and Eclipse RDF4J based on Java. Another group is inference-oriented libraries such as OWL API written in Java and Owlready2 written in Python. Both libraries support OWL. Most tools listed in Table 3. support queries.

**Table 3:** RDF APIs comparison.

API name	Programming language	Query	Reasoning	URL
Apache Jena	Java	yes	yes	https://jena.apache.org/
dotNetRDF	C#	yes	no	https://www.dotnetrdf.org/
Eclipse RDF4J	Java	yes	yes	https://rdf4j.org/
graphy.js	JavaScript	no	yes	https://graphy.link/
RDFLib	Python	yes	no	https://github.com/RDFLib/rdflib
rdflib.js	JavaScript	yes	no	http://linkeddata.github.io/rdflib.js/doc/
Redland RDF Library (librdf)	C	yes	no	http://librdf.org/
OWL API	Java	no	yes	https://github.com/owlcs/owlapi
Owlready2	Python	yes (using RDFlib)	yes	https://bitbucket.org/jibalamy/owlready2

### 2.5 Access method 3: SPARQL

SPARQL [12] queries are another method of accessing our dataset. CONSTRUCT, ASK, SELECT queries can be performed to access the MEiLD dataset. A CONSTRUCT query returns an RDF source constructed by substituting variables. An ASK query returns true or false indicating whether a query pattern matches or not. A SELECT query returns the variables bound in a query pattern match. This type of query consists of three parts in general:

1 PREFIX declares prefixes used in the query,
2 SELECT identifies the variables to appear in the query results,
3 WHERE provides the basic graph pattern to match against the date.

For example, the query

PREFIX skos: <http://www.w3.org/2004/02/skos/core#>

SELECT ?name ?nameBroader

WHERE {

?m skos:broader ?b;

skos:prefLabel ?name .

?b skos:prefLabel ?nameBroader .

} returns the name of the molecular entity and the name of the broader chemical particle. Due to links to various resources, our dataset can use federated queries, i.e. provide solutions based on information from many different sources.

### 2.6 Access method 4: Notation3 reasoning

Notation3 [13] extends RDF with variables and nested graphs to enable the declarations of rules. A rule of the form body => head has an antecedent graph to the left of the => symbol are the premise of the rule and a consequent graph to the right. For example, the rule

{

?m1 skos:broader ?m2 .

?m2 skos:broader ?m3 .

}

=>

{

?m1 ex:moreGeneral ?m3 .

} defines which chemical particles are more general. All new RDF triples deduced during the incremental reasoning are put together and merged with existing triples.

## CRediT authorship contribution statement

**Dominik Tomaszuk:** Conceptualization, Methodology, Validation, Investigation, Visualization, Writing - original draft. **Łukasz Szeremeta:** Software, Validation, Resources, Writing - original draft.

## Declaration of Competing Interest

The authors declare that they have no known competing financial interests or personal relationships which have, or could be perceived to have, influenced the work reported in this article.

## Appendix Supplementary materials

Image, application 1

Image, application 2

## Footnotes

1 https://github.com/lszeremeta/SDFEater

2 https://www.ebi.ac.uk/chebi/

3 https://creativecommons.org/licenses/by/4.0/

4 https://goessner.net/articles/JsonPath/

5 https://stedolan.github.io/jq/
