# Supplementary material for: The molecular entities in linked data dataset
Source: Data Brief. 2020 May 27;31:105757. doi: 10.1016/j.dib.2020.105757 (PMC7276506; doi:10.1016/j.dib.2020.105757)
Supplement: Supplementary file 1 [file mmc1.pdf]

Dominik Tomaszuk: Conceptualization, Methodology, Validation, Investigation, Visualization, Writing - Original Draft **Łukasz Szeremeta**: Software, Validation, Resources, Writing - Original Draft
